# Development of a multilevel intervention to increase colorectal cancer screening in Appalachia

**DOI:** 10.1186/s43058-021-00151-8

**Published:** 2021-05-19

**Authors:** Aaron J. Kruse-Diehr, Jill M. Oliveri, Robin C. Vanderpool, Mira L. Katz, Paul L. Reiter, Darrell M. Gray, Michael L. Pennell, Gregory S. Young, Bin Huang, Darla Fickle, Mark Cromo, Melinda Rogers, David Gross, Ashley Gibson, Jeanne Jellison, Michael D. Sarap, Tonia A. Bivens, Tracy D. McGuire, Ann Scheck McAlearney, Timothy R. Huerta, Saurabh Rahurkar, Electra D. Paskett, Mark Dignan

**Affiliations:** 1 University of Kentucky College of Public Health, Lexington, KY USA; 2 University of Kentucky Markey Cancer Center, Lexington, KY USA; 3 The Ohio State University Comprehensive Cancer Center, Columbus, OH USA; 4 The Ohio State University College of Public Health, Columbus, OH USA; 5 The Ohio State University College of Medicine, Columbus, OH USA; 6 Northeast Kentucky Area Health Education Center, Morehead, KY USA; 7 Ohio Hills Health Services, Quaker City, OH USA; 8 Southeastern Med, Cambridge, OH USA; 9 Lewis County Primary Care Center, Inc. dba PrimaryPlus, Vanceburg, KY USA

**Keywords:** Colorectal cancer, Multilevel interventions, Appalachia, Implementation

## Abstract

**Background:**

Colorectal cancer (CRC) screening rates are lower in Appalachian regions of the United States than in non-Appalachian regions. Given the availability of various screening modalities, there is critical need for culturally relevant interventions addressing multiple socioecological levels to reduce the regional CRC burden. In this report, we describe the development and baseline findings from year 1 of “Accelerating Colorectal Cancer Screening through Implementation Science (ACCSIS) in Appalachia,” a 5-year, National Cancer Institute Cancer Moonshot^SM^-funded multilevel intervention (MLI) project to increase screening in Appalachian Kentucky and Ohio primary care clinics.

**Methods:**

Project development was theory-driven and included the establishment of both an external Scientific Advisory Board and a Community Advisory Board to provide guidance in conducting formative activities in two Appalachian counties: one in Kentucky and one in Ohio. Activities included identifying and describing the study communities and primary care clinics, selecting appropriate evidence-based interventions (EBIs), and conducting a pilot test of MLI strategies addressing patient, provider, clinic, and community needs.

**Results:**

Key informant interviews identified multiple barriers to CRC screening, including fear of screening, test results, and financial concerns (patient level); lack of time and competing priorities (provider level); lack of reminder or tracking systems and staff burden (clinic level); and cultural issues, societal norms, and transportation (community level). With this information, investigators then offered clinics a menu of EBIs and strategies to address barriers at each level. Clinics selected individually tailored MLIs, including improvement of patient education materials, provision of provider education (resulting in increased knowledge, *p* = .003), enhancement of electronic health record (EHR) systems and development of clinic screening protocols, and implementation of community CRC awareness events, all of which promoted stool-based screening (i.e., FIT or FIT-DNA). Variability among clinics, including differences in EHR systems, was the most salient barrier to EBI implementation, particularly in terms of tracking follow-up of positive screening results, whereas the development of clinic-wide screening protocols was found to promote fidelity to EBI components.

**Conclusions:**

Lessons learned from year 1 included increased recognition of variability among the clinics and how they function, appreciation for clinic staff and provider workload, and development of strategies to utilize EHR systems. These findings necessitated a modification of study design for subsequent years.

**Trial registration:**

Trial NCT04427527 is registered at https://clinicaltrials.gov and was registered on June 11, 2020.

Contributions to the literature

- We describe the formation of a multicomponent multilevel intervention (MLI) in rural Appalachian primary care clinics.
- Pilot year findings informed strategies and adaptations that will be implemented during years 2 through 5 of the multiyear intervention.
- Lessons learned from the first year of ACCSIS Appalachia can inform other researchers seeking to implement evidence-based colorectal cancer screening programs in low-resource, rural settings.

## Background

Colorectal cancer (CRC) is a significant public health problem in several areas of the United States (U.S.), including the Appalachian region [1]. Appalachia is a 13-state, 205,000-square-mile region that ranges from the southernmost counties in New York to northeastern Mississippi [2]. While the region primarily follows the Appalachian mountain range and is thus geographically defined, the Appalachian Regional Commission has expanded the federal designation of Appalachia to include more distal locations, such as northeastern Ohio and Mississippi, based on similar economic concerns and a need to direct increased funding to non-core Appalachian counties [3, 4]. More than 25 million people live in Appalachia, where almost half of the area is rural and most of the residents are White and non-Hispanic [5]. The Appalachian region has higher than average incidence [5, 6] and mortality rates for CRC, with Kentucky and Ohio experiencing some of the highest rates in the nation [7]. Appalachian residents are more likely to have lower incomes, higher poverty rates, lower levels of education, higher unemployment rates, and poorer health than non-Appalachian residents [4]. Given the significant socioeconomic disparities across this geographically unique, medically underserved region [1, 4], there is critical need for unique, culturally relevant interventions to reduce the burden of CRC in Appalachia.

Although screening tests for CRC (e.g., colonoscopy, sigmoidoscopy, stool tests) that can reduce both incidence and mortality from CRC have been available, CRC screening prevalence is lower in Appalachian regions of Kentucky and Ohio as compared to non-Appalachian regions [8]. Low CRC screening rates in Appalachia are associated with intrapersonal and health care provider-related factors, but are also linked to state and community factors such as access to health care and poverty [9–11]. Factors such as high poverty rates, cultural and religious considerations, and isolating geographical characteristics [12] in rural Appalachia make safety net clinics, health care providers, and community resources critical sources of preventive care. Appalachians, in general, live in tightly knit, rural communities where information from non-local sources is carefully evaluated to determine how it could be applied in the community [13]. Though providers and health care delivery systems have clear roles in recommending and providing access to CRC screening, increased screening rates are also reliant on positive community norms and patient factors such as improved CRC knowledge and allaying fears surrounding the screening tests [14, 15]. Thus, to engage Appalachian populations, information needs to be presented via multiple channels as well as in ways and through trusted sources that demonstrate understanding of local interests, values, and communication styles [16].

Multicomponent interventions that use a combination of two or more approaches in three strategic areas—increasing community demand, increasing community access, and improving provider delivery of screening services—have been found to be effective in increasing CRC screening [17]. However, factors such as low perceived risk, lack of knowledge about the need for screening and screening guidelines, belief that screening is not necessary, provider distrust, and negative emotional perceptions about screening such as embarrassment or fear have each shown to be major patient-level barriers to CRC screening in Appalachian populations [18–20]. At the clinic level, barriers to receipt of screening include lack of comprehensive educational materials and insufficient tracking systems to remind clinic staff to follow-up with patients in need of screening [18]. Furthermore, Appalachians have frequently encountered barriers such as lack of physician recommendation as a reason for having never received CRC screening [19], and medical professionals identified limited time and high patient volume as barriers to providing CRC screening, along with acute medical concerns and procedural/reimbursement issues [10, 15, 19]. Lastly, low rates of educational attainment combined with fatalistic beliefs and fear of cancer may contribute to the lack of discussion surrounding CRC and screening [21]. Clearly, to reduce CRC screening disparities, interventions must address barriers at multiple socioecological levels [22], e.g., patient, provider, clinic and community, or multilevel interventions (MLIs).

Researchers at the University of Kentucky (UK) and Ohio State University (OSU) have conducted prior research in Appalachia addressing each of these levels of influence across Appalachian Kentucky and Ohio. From 2003 to 2018, they implemented the “Appalachia Community Cancer Network,” a National Cancer Institute (NCI)-funded effort to address cancer disparities in the region that included, among other programs, a faith-based initiative to decrease cancer burden in rural Kentucky and Ohio by reducing obesity and increasing cancer screening rates [23, 24]. Investigators at both institutions have also implemented projects to reduce CRC with an emphasis on clinic-level [10, 25–28], provider-level [10], patient-level [29], and community-level [30–33] factors. Although each project provided new information about cancer control in Appalachia, the projects also pointed to the need to provide interventions that could simultaneously intervene at multiple levels of influence rather than address individual levels one at a time. This revised implementation approach, therefore, guided development of the current project, Accelerating Colorectal Cancer Screening through Implementation Science in Appalachia (ACCSIS Appalachia), one of five Accelerating CRC Screening and Follow-up through Implementation Science (ACCSIS) programs funded by the NCI’s Cancer Moonshot^SM^ initiative promoting acceleration of cancer research.

The overall aim of ACCSIS is to conduct multi-site, coordinated, transdisciplinary research to evaluate and improve colorectal cancer screening processes using implementation science strategies. In this report, we describe the developmental process and year 1 pilot findings for ACCSIS Appalachia and how we plan to test our approach in a randomized controlled trial in 10 additional counties in Appalachian Ohio and Kentucky during years 2–5.

## Methods

ACCSIS Appalachia was developed in two phases, and all components of the project were approved by the institutional review board of The Ohio State University prior to engaging in project activities. Phase I focused on establishing the project and conducting activities needed to develop and test an MLI. These activities were conducted in one Appalachian county in Kentucky and one county in Appalachian Ohio and included a series of activities: (1) identifying and describing the study communities and primary care clinics; (2) planning the MLI; and (3) conducting a pilot test of the MLI using customized clinic-specific implementation strategies for EBIs at each level. As an MLI project, the focus is on the patient, provider, clinic, and community levels, with intervention delivery mainly occurring within the primary care clinic setting and community-level strategies often being supported by clinic staff and efforts. The main outcomes from ACCSIS are CRC screening, follow-up of abnormal tests, and referral to care; data to assess outcomes will come from the electronic health record (EHR) systems of the participating primary care clinics. Secondary outcomes include community-level assessment of self-reported CRC screening collected from serial random-digit-dialed telephone surveys. During year 1 activities, our primary foci included assessment of factors (i.e., facilitators and barriers) affecting implementation of evidence-based interventions (EBIs) and pilot testing our research strategy for refinement prior to full project trial implementation during Phase II in years 2–5.

### Theoretical framework

**Fig. 1:**
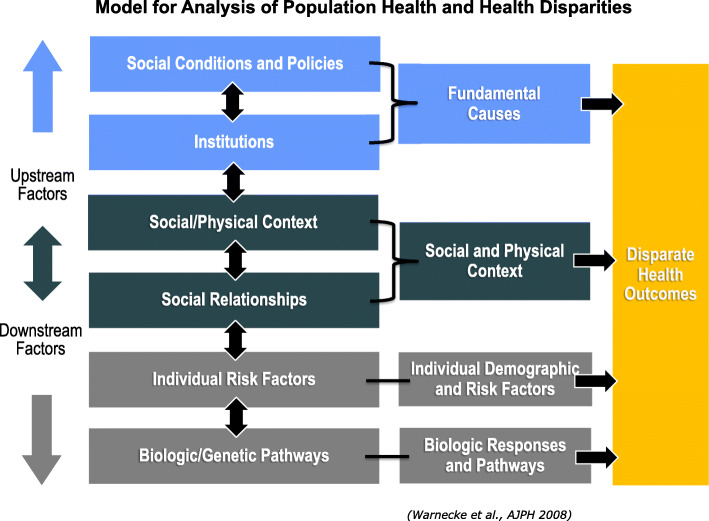
Model for analysis of population health and health disparities

Development of the ACCSIS project was guided by three theoretical frameworks. First, the Social Determinants of Health Model provided the overall conceptual foundation for the project. Second, the Model for the Analysis of Population Health and Health Disparities, developed by the Center for Population Health and Health Disparities (CPHHD), was used to understand not only the multilevel factors that hinder and facilitate adherence to CRC screening recommendations, but help understand the relationships between levels [34]. As shown in Fig. 1, the model focuses attention on factors termed “‘upstream” and “downstream” that contribute to health disparities. This conceptual approach is particularly useful in planning disparity-reducing interventions as it focuses attention on community-level resources that could play roles in reducing disparities as well as clinic- and individual-level characteristics that may or may not be modified by interventions.

Third, a model described by Proctor et al. [35] that guides the implementation and evaluation of EBIs was used to develop ACCSIS Appalachia. Specifically, implementation (e.g., fidelity, feasibility, acceptability and sustainability), service (e.g., effectiveness), and client (e.g., satisfaction) outcomes will be assessed throughout the implementation period. Data collection to measure these outcomes is ongoing, and results will be reported in a future manuscript. Application of each of these models was guided by principles of Community-Based Participatory Research (CBPR). CBPR provides a foundation that focuses on including the community experiencing disparities as a partner. Through CBPR, investigators are led to understand disparities from the point of view of the community and to consider intervention strategies that the community wants and needs to address health disparities.

### Study population

Data for 2010–2014 showed that, compared to non-Appalachian counties, Appalachian counties of Kentucky and Ohio had higher CRC incidence (14.5% and 3.9% difference, respectively) and mortality (26.4% and 10% difference, respectively) [8]. With support from the Centers for Disease Control and Prevention, each state conducts annual telephone surveys of health behaviors. This survey is referred to as the Behavioral Risk Factor Surveillance System (BRFSS) survey. Data from the BRFSS surveys in 2014 and 2016 for Kentucky and Ohio showed that screening rates were higher in non-Appalachian counties, with the exception of stool blood testing in Kentucky [8].

**Fig. 2:**
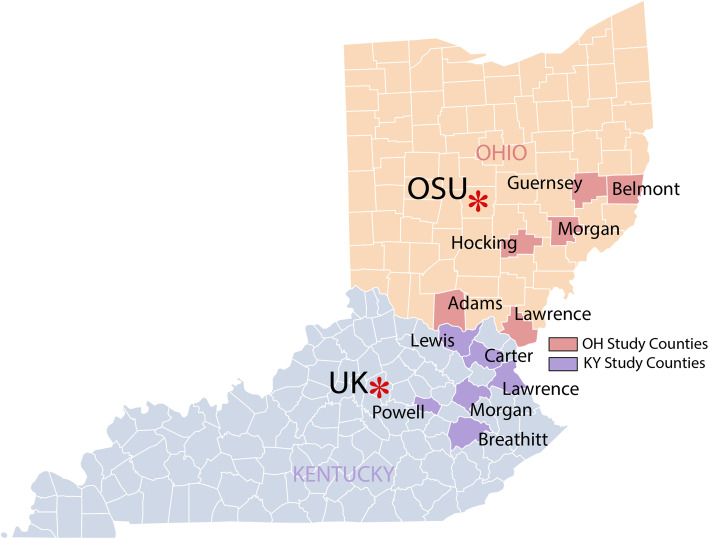
Participating ACCSIS counties

We selected 12 counties, six in Appalachian Kentucky and six in Appalachian Ohio, to participate in ACCSIS in Appalachia (Fig. 2). The counties were selected based on a thorough review of cancer registry and BRFSS data, socioeconomic indicators, and characteristics of primary care clinics to ensure that counties (a) were representative of areas of central Appalachia that experience cancer health disparities and high CRC incidence and mortality rates and (b) had clinics that were willing and able to engage in research, a determination facilitated by conversations between individual clinics and our project partners at the Northeast Kentucky Area Health Education Center (AHEC). To capture data about clinic and county CRC burden, community and patient socio-demographic profiles, and available health system and community health resources, we carried out a multilevel assessment in each county. Activities included conducting key informant interviews, creating community profiles, identifying clinic and community champions, and performing health clinic data inventories (e.g., baseline group-level CRC screening and follow-up rates, EHR capabilities, staff roles). Descriptive characteristics of the study counties are provided in Table 1 and characteristics of the project clinics are shown in Table 2. As shown, the population of the ACCSIS Appalachia counties experience low incomes and suboptimal CRC screening rates that are lower than state and national rates.

**Table 1:** Colorectal cancer data, screening, and demographic characteristics of the counties in the study population

	Incidence^**b**^	Mortality^**b**^	% Screened^**c**^	% Female^**d**^	# age 45-74 (%)^**e**^	Median household income^**f**^	% Below poverty (age 18–64)^**g**^	% Below poverty (age 65+)^**g**^
US	125.1	48.9	66.3	50.8	103,202,874	$55,322	14.2	9.3
Kentucky	156.9	57.3	67.2	50.8	1,507,404 (35%)	$44,811	18.0	11.4
Breathitt	183.0	76.8	54.5	50.0	5225 (38%)	$25,484	34.1	17.2
Carter	150.7	83.2	64.6	50.4	4281 (37%)	$35.095	28.2	10.0
Lawrence	215.0	111.9	58.4	50.6	6024 (38%)	$35,016	22.6	19.3
Lewis	158.6	78.7	61.7	50.1	5221 (38%)	$29,088	27.2	20.1
Morgan	126.5	^a^	53.4	43.3	4910 (35%)	$32,517	20.6	24.9
Powell	261.9	^a^	62.9	50.3	4564 (36%)	$34,048	25.8	15.5
Ohio	130.0	54.3	65.1	51.2	4,044,691 (35%)	$50,674	14.7	8.1
Adams	174.3	84.7	64.3	50.6	10,396 (36%)	$34,709	24.8	10.9
Belmont	111.8	65.7	62.7	49.6	27,495 (39%)	$44,719	14.6	8.6
Guernsey	176.4	74.0	63.2	51.0	15,090 (38%)	$41,566	19.0	8.6
Hocking	136.9	84.5	59.3	50.0	11,321 (39%)	$43,382	15.9	8.7
Lawrence	141.7	61.2	64.7	51.4	22,926 (37%)	$44,256	17.6	12.1
Morgan	126.2	74.2	64.5	50.2	5867 (39%)	$38,941	19.9	11.9

^a^ Suppressed due to small number of cases

^b^https://statecancerprofiles.cancer.gov/, per 100,000 population, 2010–2014, age 50+

^c^https://statecancerprofiles.cancer.gov/, per 100,000 population, 2008–2010, age 50+

^d^https://www.socialexplorer.com/, 2010 (US Census Bureau)

^e^https://factfinder.census.gov/faces/nav/jsf/pages/index.xhtml, 2010 (US Census Bureau)

^f^https://factfinder.census.gov/faces/nav/jsf/pages/searchresults.xhtml?refresh=t, in 2016 inflation adjusted dollars (American Community Survey)

^g^https://factfinder.census.gov/faces/nav/jsf/pages/searchresults.xhtml?refresh=t, 2012–2016 (American Community Survey)

**Table 2:** Characteristics of project clinics

Clinic counties	Clinic type	Clinic size^**a**^	% Medicaid	# Providers^**b**^	RUCA	EHR
Ohio						
Guernsey (Pilot)	FQHC	396	21.5	4	5	eClinicalWorks
Adams	FQHC	496	1.2	2	3	NextGen
Belmont	FQHC	437	29.8	2	1	NextGen
Hocking	FQHC	2641	17	6	7	eClinicalWorks
Lawrence	FQHC	2837	43	4	1	eClinicalWorks
Morgan	FQHC	N/A	47	3	6	Epic
Kentucky						
Lewis (Pilot)	FQHC	1746	35	9	10	Athena
Lawrence	Regional system	1449	32	4	2	Athena
Morgan	Regional system	1654	29.2	4	10	Meditech
Breathitt	FQHC	1061	54.1	7	8	Allscripts Pro
Carter	Regional system	950	46	4	2	Meditech
Powell	Regional system	558	31	4	5	Epic/Explorys

*N/A* not available

^a^ # adults age 50–74; ^b^ includes full-time physicians, part-time physicians, nurse practitioners, and physicians’ assistants

### External scientific advisory board

The research team identified five experts from outside institutions with experience in community-based cancer control research, CRC screening interventions, implementation science, and/or rural or Appalachian health to participate in an external scientific advisory board (ESAB) and provide input on project development and implementation. ESAB members were provided with the project protocol and data collection tools to review, and they provided the ACCSIS team with recommendations and suggested subsequent steps.

### Community advisory board

Additionally, the ACCSIS team recruited community advisory board (CAB) members from the identified project counties in KY and OH. Participants were identified via word of mouth, clinic recommendations, and key informant interviews. Members include clinic and community champions, community leaders, health department and cooperative extension representatives, and community health workers.

### Formative clinic and community evaluation

After incorporating ESAB and CAB suggestions, investigators conducted key informant interviews with community members and clinical stakeholders to help develop a locally relevant menu of EBIs from which pilot clinics could select MLIs. Informed consent was obtained from each key informant who completed a brief demographic survey prior to beginning the discussion. Interviews were audio-recorded and supplemented by handwritten notes. For the community and clinical key informant groups, the research team created a coding spreadsheet, identifying high-level themes and overarching findings for each broad category in the respective interview guide. Investigators conducted interviews in-person, via telephone, or via web-conferencing. Semi-structured interview guides, informed by a review of the literature and the research team’s prior experience in Appalachia, were used to organize the interview discussions with the community members and clinical stakeholders, respectively. Interview guides concentrated on a SWOT (Strengths, Weaknesses, Opportunities, and Threats) analysis for CRC screening at the patient, provider, clinic, and community levels.

Community key informants were recruited using multiple strategies including personal email invitations and referrals from existing partners (e.g., members of local cancer coalitions). Recruited community members represented local agencies committed to serving area residents, including cooperative extension, community health worker programs, local health departments, hospitals, and cancer coalitions. The interview guide for community members focused on perceptions of their community’s health, socioeconomic status, culture, CRC awareness, personal and community knowledge of CRC screening tests, barriers to CRC screening in their communities, community assets and/or programming that could be leveraged to promote CRC screening, and preferences for messaging and channels for delivery of community-based CRC screening education.

Clinical stakeholders were individuals from participating health centers including doctors, nurses, certified medical assistants, administrators, quality improvement coordinators, and front desk staff. Similarly, the interview guide for clinical stakeholders asked about perceptions of community- and patient-oriented challenges to CRC screening, clinic- and provider-related practices (e.g., patient education, recommended screening tests, reminder systems, patient tracking, delivery of screening results), and preferences for patient, provider, and community messaging and formats for CRC screening education.

Phase I activities occurred during year 1 of the project with Phase II activities scheduled for years 2 through 5. Phase I activities were pilot tested in one Appalachian Kentucky and one Appalachian Ohio county. Phase II activities will include review and revision of the MLI approach tested in Phase I and evaluation of effectiveness in a group randomized trial implemented in five additional counties in Appalachian Kentucky and five additional counties in Appalachian Ohio. Results from Phase II will be reported in a future publication.

## Results

### Formative evaluation

A total of 12 CAB members in Kentucky and 10 in Ohio were recruited to the project, and to date, we have held four CAB meetings: in January 2019 (Kentucky), March 2019 (Ohio), May 2019 (Kentucky), and August 2019 (Ohio). Meetings provided CAB members with the opportunity to offer input and recommendations on the proposed objectives and processes of Phase I of ACCSIS Appalachia. Topics for CAB meetings included an overview of the initiative and themes reflected in key informant interviews, summary of clinic and community intervention activities, and requests for feedback on telephone survey materials, as well as suggestions for increasing community participation and engagement at future events.

### Key informant interviews

We conducted interviews with 24 community members (*n* = 13 in Kentucky, *n* = 11 in Ohio) and 51 clinical stakeholders (*n* = 20 in Kentucky, *n* = 31 in Ohio) in the 12 intervention counties between February and May 2019. On average, the interviews took 45–60 min. Key informant interviews identified strengths such as providers encouraging CRC screening among patients and positive and strong community connections, among others. Common CRC screening barriers were described at the (A) patient level (e.g., serious competing priorities, fear of screening procedure and test results, financial concerns); (B) provider level (e.g., time, competing priorities); (C) clinic level (e.g., lack of reminder or tracking system, staff burden); and (D) community level (e.g., cultural issues, societal norms, transportation) [14, 36]. Opportunities included having a prevention-focused message with CRC screening options and using acceptable communication channels (e.g., social media, faith community, low literacy print materials) that would leverage the importance of staying healthy for individuals’ families. Lastly, reported threats included negative anecdotal stories, loss of health insurance due to unemployment related to a cancer diagnosis, patient issues related to insurance copayments, incongruence of screening modality suggestions by providers, and ongoing clinic issues with EHR capabilities.

Ultimately, the qualitative findings guided the research team’s decision to create a menu of multilevel (i.e., provider/clinic, patient, community), evidence-based strategies for clinics to choose from to implement in the year 1 pilot. In addition, pilot community events held in year 1 focused on provision of CRC and screening education in a creative and engaging manner at a well-regarded community location. For example, in Kentucky, the ACCSIS team partnered with a federally qualified health center (FQHC) and cooperative extension to host a community luncheon that included an expert speaker (i.e., nurse practitioner) and tours of an inflatable colon. Subsequent discussions of community-level approaches focused on the need to raise community awareness via multiple communication methods, including health fairs and mass communication mediums (e.g., newspapers, billboards), that CRC is both preventable and treatable.

### Pilot project implementation

After formative evaluation activities were complete, year 1 pilot project activities commenced in two clinics/communities (i.e., one in Kentucky and one in Ohio). Project staff and clinic champions conducted in-person implementation meetings with clinic staff and providers, project team members, and any other interested stakeholders with the goal of reviewing relevant national and local CRC statistics, discussing themes identified in the key informant interviews and environmental scans, reviewing baseline CRC screening rates based on EHR reports, describing the levels of implementation on which to focus, and highlighting possible strategies for each level. In Ohio, the pilot clinic chose patient education (patient level), provider education (provider level), and improving EHR reports and alerts and creating a written pathway to care for CRC screening (clinic level) as strategies to improve CRC screening in their patient population. In Kentucky, the pilot clinic champion, in consultation with clinic staff, selected patient education and fecal immunochemical testing (FIT) reminders/follow-up (patient-level), feedback/assessment and provider education (academic detailing), and developing a screening protocol and improving EHR reporting to increase annual wellness visits (AWVs) (clinic level). Specific details about each intervention level are displayed in Table 3 and described in detail below.

**Table 3:** Multilevel project activities in pilot counties

	Guernsey (OH)—pair 1	Lewis (KY)—pair 1
**Patient level**	• Telephone reminders for CRC screening (mailed FIT or ordered Cologuard)	• FIT reminder postcards
		• Screen for Life education
		• FIT/Cologuard provider video
	• Provider letter with CRC Screen for Life pamphlet	
**Provider level**	• Assessment with feedback	• Assessment with feedback
	• Provider education	• Provider education
	• One-on-one call with gastroenterologist for education, encouragement, provider barrier counseling	
**Clinic level**	• EHR alerts	• CRC risk assessment added to EHR
		• Annual Wellness Visit reminders/incentives
**Community level**	• Community outreach events to promote screening—health fair; farmer’s market; inflatable colon	• Community CRC screening billboards
		• Community outreach events to promote screening—community luncheon; inflatable colon

### Patient-level interventions

The Ohio pilot clinic implementation team chose Healthy Colon, Healthy Life as the patient-level EBI to implement in their clinic because it offered the potential to reach those patients who do not present in clinic at regular intervals [37]. Components included the following: (1) a phone call from clinic staff to the patient to confirm eligibility, identify stage of change, and provide barrier counseling for CRC screening (i.e., counseling about how to reduce barriers to screening); (2) a mailed fecal occult blood test (FOBT) accompanied by CRC screening educational brochure and a letter from their provider encouraging them to complete the at-home screening test; and (3) a follow-up phone call from clinic staff to the patient if the FOBT was not returned for processing. Adaptations implemented by clinic staff included using the FIT instead of the FOBT and using a Centers for Disease Control and Prevention (CDC) Screen for Life brochure instead of the educational brochure used in the sentinel study. Clinic staff also opted to display CDC Screen for Life posters in their clinic space to facilitate conversation around CRC screening and begin to increase knowledge of the importance of CRC screening. Similarly, the Kentucky clinic chose to provide patient education using Screen for Life materials. They chose to focus their patient-level education on improving distribution and return of FIT. Specifically, they mailed FIT reminder birthday postcards to patients due for annual FIT, and a clinic nurse created a demonstration video on how to complete FIT and Cologuard, which was subsequently publicized on the clinic’s social media pages.

### Provider-level interventions

At the provider level, the Ohio clinic chose to implement a provider education EBI that focused on follow-up of abnormal CRC screening tests, consistent with the clinic’s goal of 100% follow-up [38]. Components included two small group provider education sessions on CRC and barriers to complete diagnostic evaluation (offered 6 months apart), a pre-post survey to assess change in knowledge, printed provider education materials, a tailored letter and phone call from the trainer to the provider, a practice-specific report about CRC screening, and an education session evaluation. The clinic adapted this EBI to focus provider education not only on follow-up of abnormal CRC screening, but also education about strategies to increase initial CRC screening. The education was provided by OSU researchers who conducted the hour-long session at the clinic during the clinic lunch break (September 2019). Eight clinic/health system providers and staff members attended. Pre- and post-knowledge data were obtained from the providers: prior to the education session, they averaged 15.3 ± 1.8 CRC knowledge questions correct out of 20 which improved to an average of 18.4 ± 1.5 correct following the education session (*p* = 0.003).

Similarly, an intervention kickoff event was held by UK researchers in November 2019 to present clinic staff details of their chosen EBIs, which included scheduled biannual expert speakers to provide detail on high-level, pertinent CRC screening topics for providers. The kickoff presentation concluded with an educational session to assist providers with motivating patients to complete CRC screening. However, in previous discussions, the clinic indicated that provider knowledge of screening modalities and guidelines was excellent and should not be the focus of any provider education EBI sessions. Citing patient adherence as a primary factor in low screening rates, the Kentucky clinic chose to implement a provider education EBI that focused on communication strategies providers could utilize to motivate both willing and reluctant patients to complete CRC screening. The education session was provided by UK researchers with expertise in CRC research and communication strategies.

### Clinic-level interventions

The pilot clinics in Kentucky and Ohio selected clinic-level EBIs they felt would increase CRC screening and were best suited to their needs. The Ohio clinic fine-tuned their use of eClinicalWorks (eCW) to identify patients in need of CRC screening and follow-up and created a written pathway to clinical care for CRC screening and follow-up of abnormal tests. The written pathway documented the combination of the chosen EBIs and how their respective components were assimilated into their existing clinic operations. It also specified the process of identifying patients eligible for screening, ensuring that patients received a CRC screening recommendation (either in-person or via mail), tracking completion of CRC screening in eCW and referral follow-up, monitoring progress, and making improvements, as needed.

The Kentucky clinic also developed a CRC screening protocol to ensure implementation fidelity, and they decided to focus on increasing the number of patients who visit the clinic for annual wellness visits (AWV). In making this decision to leverage AWVs to increase CRC screening rates, the clinic pointed out the limited time that providers have to discuss CRC with patients during acute care visits, and also that AWVs are underutilized by patients. AWVs provide the best opportunity and time for providers to discuss CRC and other screenings with patients in detail and use motivational communication techniques to encourage patients to follow through and complete screening. To implement this strategy, the clinic decided on a two-pronged approach: (1) at the patient level, the clinic will send birthday card reminders to patients encouraging them to schedule AWVs; and (2) at the provider/clinic level, the clinic will set goals and provide incentives for providers and staff who increase their respective AWV numbers.

### Community-level interventions

The pilot clinics in both states chose to use the “inflatable colon” [39] to educate community members about polyps, potential symptoms, and the importance of regular CRC screening. In Guernsey County, Ohio, the pilot clinic partnered with a local regional medical center to raise awareness about CRC and related screening [40]. Between May and July 2019, an inflatable walk-through colon exhibit was set up at two community events including a local farmer’s market and a festival. This interactive format included guided tours of the colon exhibit by health care professionals to educate 70 lay community members about prevention and early detection of CRC. The participants received a card with CRC cancer screening recommendations and contact information for free or reduced cost screening available in their county. Local media coverage of these events included local radio station and newspaper, along with a video of a guided tour of the colon exhibit posted on Facebook.

A similar event was held in Lewis County, Kentucky, in May 2019. The outreach event was held in conjunction with the local county extension office. The event included a presentation from a clinic provider on how to complete FIT and Cologuard tests, a healthy cooking demonstration, a testimonial from a community member about her personal colonoscopy experience, and guided tours of the inflatable colon. Forty-six community members attended the event. Additionally, to help reach patients who may not visit the clinic regularly, members of the Kentucky CAB in the pilot county decided to use mass media to deliver a message that CRC is “preventable, treatable, and beatable.” To that end, a billboard was collaboratively designed with input from Kentucky ACCSIS project team members, clinic staff, and university marketing staff. The billboard was erected in December 2019 in a high-visibility location along a main highway, and it included the aforementioned simple message; the logos of the university, project, and local clinic; a contact phone number for community members to inquire about screening; and a photo of a known practitioner from the clinic.

### Study design modification

Throughout the course of these multilevel program activities, it became apparent that supporting clinics throughout EBI implementation requires more resources than we initially anticipated. We originally planned on evaluating our MLI using a group randomized trial. Two counties per state would be randomized to an early intervention arm, and two counties would be randomized to a delayed intervention arm which would receive our MLI 12 months later. Counties in each study arm would experience a 12-month active intervention period and then be observed for sustainability of the MLI for either 12 or 24 months depending on the arm. However, based on our experience in the pilot year, the design was altered to introduce the intervention in a staggered fashion (Table 4).

**Table 4:** Intervention timeline for each state

		5/2019	10/2019	5/2020	10/2020	5/2021	10/2021	5/2022	10/2022	5/2023
**Pair 1**	**County 1**^**a**^	AI	AI	AI/S	S	S	S	S	S	S
	**County 2**	C	C	AI	AI	AI/S	S	S	S	S
**Pair 2**	**County 3**		AI	AI	AI/S	S	S	S	S	S
	**County 4**		C	C	AI	AI	AI/S	S	S	S
**Pair 3**	**County 5**					AI	AI	AI/S	S	S
	**County 6**					C	C	AI	AI	AI/S

*AI* active intervention period, *C* control period, *S* sustainability period, *AI/S* AI ends and *S* begins

^a^Pilot

**Fig. 3:**
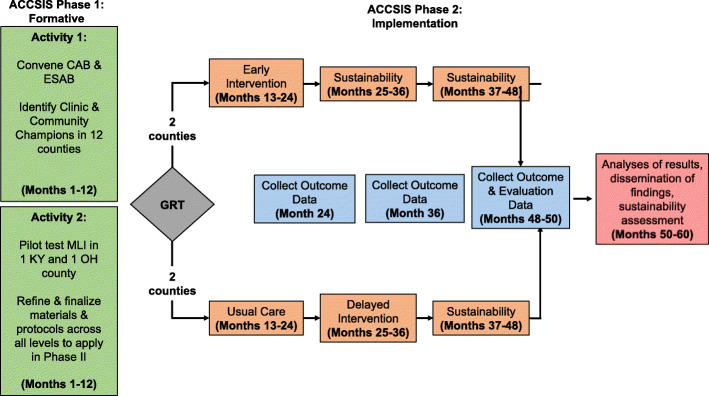
Study schema

Within each state, counties will be paired according to patient volume of the participating clinics in each county. The pair containing the pilot county (pair 1) will be the first to receive the intervention with the order of the other pairs (pairs 2 and 3) randomly determined. Study group (early, delayed intervention) will also be randomly assigned within pairs 2 and 3. Thus, across the two states, we will have eight counties that will be randomized to study group and four whose group assignment will not be randomly determined. Figure 3 provides a detailed look at the study timeline in pair 1. The county assigned to the early intervention group will receive the MLI at the beginning of phase II and the other county will receive the program 12 months later (delayed group). At 12 months (end of the Phase I) and 24 months (end of Active Implementation in the Early Intervention group), we will obtain clinic-level and county-level CRC screening rates using the EHRs and county-level behavioral assessment survey, respectively. In addition to at 12 and 24 months, screening rates will be collected at baseline (i.e., month 1), and 36- and 49-month time periods to assess trends. The timelines for pairs 2 and 3 are similar, though the sustainability periods are shorter since the MLI is introduced later in the study. To minimize potential contamination from community activities, we intentionally selected project counties that were either non-adjacent or that had unique travel patterns (i.e., common cities where residents were likely to seek out services such as restaurants, activities, and medical care) that did not overlap with other study counties. Furthermore, we plan to collect survey data from control clinics at the end of the intervention to account for any potential contamination among residents of different project counties.

Lastly, our pilot work indicated that a change in primary outcomes was necessary. Originally, our primary outcome was the county-level CRC screening rate which would be determined using telephone surveys conducted every 12 months in each county. However, most of the intervention activities occurred in the clinics, not in county-wide community settings, so assessing county-level CRC screening rates did not seem to reflect the effects of our partners’ greatest efforts. Given our study design, we would expect stronger effects on clinic-level rates than county-level rates which may demonstrate lagged effects due to the time required for the intervention to permeate from the clinic to the community. We also realized during the pilot period that in order to reach our target sample size of 100 participants per county, data collection required more time than anticipated and would span both the active intervention and sustainability period in the early intervention arm as well as the control and active intervention periods in the delayed intervention arm, which is not ideal for estimating an intervention effect. As a result, we changed our primary outcome to clinic-level CRC screening rates determined via EHR. Since exact screening dates are available in the EHR, we can easily determine the number of patients screened during the control, active intervention, and sustainability periods.

## Discussion

In this report, we have described our experiences during year 1 of a 5-year project that will deliver an MLI to increase CRC screening and follow-up in underserved areas of the U.S., specifically Appalachian Kentucky and Appalachian Ohio. Preliminary tasks included selecting rural primary care sites in Appalachian counties and developing relationships to serve as a foundation for implementing the program. A theory-guided approach used by the investigators focused on careful assessment of community and clinic assets and barriers that would influence intervention implementation. The structure and management of the primary care clinics, the EHRs that they use, and community context were important drivers of progress in developing the project. The pilot phase proceeded as planned and provided important information on how to move forward with the full-scale trial.

A strength of the process was that the investigators have extensive history of working in partnership with Appalachian communities [10, 23–33] and have thus developed sustained, trusting relationships. This history has included extensive travel and face-to-face meetings with local residents where cultural nuances were made clear. For example, through these meetings, the investigators learned about sensitivity to cultural stereotypes and assumptions that are often made about the region and its people. Gaining an understanding of the culture, issues experienced by the communities, and barriers clinics in these regions are facing contributed to the success of the pilot phase. The information gathered in piloting the intervention approach in two clinics informed the structure and development of the full implementation of the program coming in years 2 through 5.

One of the primary lessons learned was the extent of the challenges associated with the range of administrative structures of the primary care clinics. Although community structures were relatively uniform and characteristic of rural Appalachia, the primary care clinics had distinctly different structures. Several clinics are FQHCs and others are satellite primary care clinics of regional hospitals. The satellite primary care clinics are governed by the corporate policies of the regional hospitals and have varying degrees of decision-making authority. Additionally, as health care delivery in rural areas has been in flux in recent years [41], change has occurred in the structures of health care corporations since the project began. The FQHCs, on the other hand, are independent Health Resources and Services Administration-funded entities that make their own decisions. This type of variability necessitates that researchers working in Appalachian primary care clinics be flexible with clinic partners, including encouraging clinic champions to adapt EBI strategies to reflect regional and clinical contextual needs [42, 43]. These locally relevant adaptations, in turn, can increase clinic capacity for promoting CRC screening and thus improve the likelihood of overall EBI success [44].

Finally, differences in EHRs among the clinics also presented challenges. A total of eight different EHRs are currently used among the 12 participating clinics and the clinics engaging in year 1 each had different EHRs. Further, none of the clinic partners maintain on-site technical support for their EHR. As a result, while clinics can generate pre-defined reports, they frequently have no ability to customize those reports or change the systems to facilitate the intervention. Such changes reportedly require the use of consultants who function remotely from the clinic. Thus, efforts to obtain screening data or add intervention components through the clinic EHRs were problematic and required extensive interactions with our staff, including health IT experts from the universities. Due to privacy concerns and institutional health system policies, efforts to assist clinic and health system staff with this activity were not successful. Given that underuse of EHR functionality tends to be more prevalent in rural areas [45], researchers working with rural independent primary care facilities (rather than clinics within an individual health care system) should prepare well in advance for possible technological limitations and consider methods by which they might mitigate these limitations, such as consulting with university IT resources during project planning to ensure clinics have appropriate technical assistance when necessary.

Although all counties and clinics participating in the project are in Appalachian Kentucky and Appalachian Ohio, there are important differences in each clinic and county that may affect CRC screening and follow-up of abnormal findings. Health care in the counties is provided by primary care practices and access to specialty care such as colonoscopy, surgery, and medical oncology is only available in large population centers. Thus, for most community residents, local access to CRC screening is limited to stool blood testing by FOBT, FIT, or Cologuard. We have plans to work with clinics to address these limitations**.**

## Conclusions

The experience gained through working with the pilot clinics in year 1 of the project provided significant lessons, including recognition of variability among the primary care clinics and how they function, appreciation for the workload of the providers and clinic staff, and understanding of barriers to gaining access to EHR data for evaluation. The experience also led to recognition that the resources needed to implement the intervention with fidelity required revision of the study design. Collectively, this formative research has informed project activities and implementation strategies for years 2 through 5 of ACCSIS Appalachia to ultimately address the problem of CRC disparities in Appalachia.

## Data Availability

Requested data available upon request to the corresponding author.

## Abbreviations

ACCSIS: Accelerating Colorectal Cancer Screening through Implementation Science
AWVs: Annual wellness visits
BRFSS: Behavioral Risk Factor Surveillance System
CAB: Community advisory board
CBPR: Community-Based Participatory Research
CDC: Centers for Disease Control and Prevention
CPHHD: Center for Population Health and Health Disparities
CRC: Colorectal cancer
EBI: Evidence-based intervention
eCW: eClinicalWorks
EHR: Electronic health record
ESAB: External scientific advisory board
FIT: Fecal immunochemical testing
FOBT: Fecal occult blood test
FQHC: Federally qualified health center
IT: Information technology
MLI: Multilevel intervention
NCI: National Cancer Institute
OSU: Ohio State University
UK: University of Kentucky
U.S.: United States
